# Complement updates in optic neuritis

**DOI:** 10.3389/fneur.2025.1566771

**Published:** 2025-03-26

**Authors:** Yuhong He, Kai Guo, Jifu Xin

**Affiliations:** ^1^Department of Ophthalmology, The Affiliated Hospital of Inner Mongolia Medical University, Hohhot, China; ^2^Department of Neurology, Mayo Clinic, Rochester, MN, United States

**Keywords:** optic neuritis, complement, neuromyelitis optica spectrum disorders, treatment, biomarker

## Abstract

Optic neuritis (ON) is an inflammatory condition of the optic nerve associated with demyelinating diseases like multiple sclerosis, neuromyelitis optica spectrum disorder, and myelin oligodendrocyte glycoprotein antibody-associated disease. The complement system is crucial in ON pathogenesis, driving blood-optic nerve barrier disruption, inflammation, and tissue damage. This review explores the complement activation pathways—classical, alternative, and lectin—and their roles in ON progression. Key proteins such as C3, C5, and terminal pathway components are highlighted as central to disease mechanisms. Recent advances in complement-targeted therapies, including C1q blockers, C3 and C5 inhibitors, show promising results in clinical and preclinical studies. Novel therapies, like anaphylatoxin receptor blockers and recombinant factor H, expand the treatment landscape, while plasma exchange remains vital for severe, corticosteroid-resistant cases. Challenges remain, such as ON heterogeneity, the long-term safety of complement inhibition, and the need for personalized approaches. Future studies should focus on unraveling complement-mediated mechanisms, identifying biomarkers, and refining therapeutic strategies. This review highlights the critical role of complement in ON and the latest therapeutic advances to improve patient outcomes.

## 1 Background

Optic neuritis (ON) is a common inflammatory condition of the optic nerve, often associated with demyelinating diseases such as multiple sclerosis (MS), aquaporin-4 antibody-positive neuromyelitis optica spectrum disorder (NMOSD), and myelin oligodendrocyte glycoprotein antibody-associated disease (MOGAD) (1–3). The pathological mechanisms of ON include inflammatory demyelination, characterized by the destruction of myelin and oligodendrocytes mediated by autoantibodies, complement activation, and antibody-dependent cell-mediated cytotoxicity (ADCC) (4). B cells and antibody-mediated complement activation contribute to astrocyte and myelin destruction. In addition, T-cell-mediated activation of macrophages and glial cells leads to blood–brain barrier (BBB) and neuronal damage. Inflammatory cell infiltration further exacerbates damage by releasing cytokines and enzymes that disrupt the blood-optic nerve barrier (BONB) (5, 6).

The complement system, a key component of innate immunity, comprises a cascade of soluble and membrane-bound proteins that facilitate pathogen elimination, mediate inflammation, and interact with adaptive immunity (7). In ON, complement activation contributes to axonal injury, demyelination, and subsequent neurodegeneration, making it a critical target for therapeutic intervention (8). Biomarkers such as C3a and C5a have been identified as indicators of disease activity, offering potential utility in diagnosis and monitoring (9). Moreover, complement inhibitors, especially in neuromyelitis optica spectrum disorder-associated ON (NMOSD-ON), have demonstrated promising therapeutic efficacy, further underscoring the relevance of the complement pathway in ON management (10). Despite these advances, significant gaps persist in our understanding of the precise roles and mechanisms of the complement system in ON. Recent progress in complement-targeted therapies for other neurological disorders suggests promising avenues for translational applications in ON. This review seeks to synthesize the current state of knowledge on the complement system in ON, focusing on recent discoveries, emerging therapeutic strategies, and unresolved questions. By elucidating these updates, the review aims to refine diagnostic and therapeutic approaches, ultimately mitigating visual disability and improving outcomes for affected patients.

## 2 Mechanisms of complement activation in ON

### 2.1 Classical pathway

The classical pathway, a vital component of the complement system, plays a central role in recognizing and eliminating pathogens, apoptotic cells, and abnormal molecular structures (11). Activation begins with the C1 complex, comprising C1q, C1r, and C1s, where C1q binds to immune complexes formed by immunoglobulin G (IgG) or immunoglobulin M (IgM) antibodies (12). This interaction triggers a cascade, leading to the cleavage of C4 and C2, forming the C3 convertase, which subsequently cleaves C3 into active fragments, C3a and C3b. Additionally, the Hageman factor fragment (HFf) can activate this pathway independently by interacting with C1, amplifying the response (13). In diseases like NMOSD-ON and MOGAD-ON, autoantibodies against AQP4 or myelin oligodendrocyte glycoprotein (MOG) initiate complement-dependent cytotoxicity (CDC), further activating the classical pathway and driving inflammation and tissue damage.

### 2.2 Alternative pathway

The alternative pathway is activated independently of antibodies and recognizes pathogen-associated molecular patterns (PAMPs) and damage-associated molecular patterns (DAMPs) on injured optic nerve cells (14). This activation occurs through the spontaneous hydrolysis of C3, forming C3 convertase on activator surfaces (15). The resulting C3 convertase generates a large amount of C3b, which combines with existing C3 convertase to form C5 convertase. This enzyme cleaves C5 into C5a and C5b, with multiple C5b molecules assembling into the membrane attack complex (MAC). The MAC inserts into target cell membranes, causing cell damage and amplifying inflammation. The alternative pathway likely plays a crucial role in antibody-negative optic neuritis (ON) and enhances responses initiated by other complement activation pathways.

### 2.3 Lectin pathway

The lectin pathway is activated by mannose-binding lectin (MBL) and associated serine proteases, such as MASP-2, which recognize carbohydrate patterns on microbial surfaces or damaged host cells. Studies have shown elevated MASP-2 expression in the retina and optic nerve, supporting its role in ON (16). Complement components such as C3 and MAC deposition precede retinal ganglion cell (RGC) loss and demyelination in animal models, indicating an early involvement of the lectin pathway in ON (16).

## 3 Key stages of complement activation in ON

### 3.1 C3 activation in ON

C3, a central component of the complement cascade, plays a critical role in the inflammatory processes associated with optic neuritis (ON). Its activation products, C3a and C3b, drive multiple inflammatory responses. Research has linked C3 activation to the progression of neuroinflammation and neurodegeneration. In ON, astrocytes express C3, associated with retinal ganglion cell (RGC) loss. Additionally, C3 activation products inhibit axonal regeneration and exacerbate neuronal damage (17). Studies have shown that reducing C3 activation mitigates RGC loss, limits axonal damage (18), and slows the progression of neurodegenerative diseases (19). The C3a receptor is also implicated in promoting neuroinflammation, with evidence suggesting that its absence reduces neurodegeneration risk, highlighting the therapeutic potential of targeting this receptor. Meanwhile, C3b acts as an opsonin, marking cells for phagocytosis and amplifying the complement cascade (20).

### 3.2 C5 activation in ON

C5 activation is integral to the classical complement cascade and contributes significantly to the pathology of ON. Antibodies targeting aquaporin-4 (AQP4) on astrocytes initiate this cascade, resulting in granulocyte, eosinophil, and lymphocyte infiltration, which damages astrocytes and oligodendrocytes, leading to demyelination and neuronal loss (21). Research shows that loss of C5a receptors can modulate NMDAR activity, reducing excitotoxicity from glutamate and mitigating neurotoxicity, thereby influencing neuromyelitis optica (NMO) pathology (22). Elevated levels of the terminal complement complex sC5b-9 in NMO patients, compared to other neurological disorders and MS, are associated with disease relapses and increased disability, highlighting its role in disease severity and progression (23).

### 3.3 C4 activation in ON

C4 is a critical component of the classical complement pathway, acting upstream in the cascade, leading to the formation of the MAC. The activation of C4 triggers a series of downstream reactions, ultimately resulting in MAC formation, which disrupts cell membranes and causes cell damage and death. Studies suggest that low C4 levels are associated with specific clinical features of NMO, including more severe BBB disruption and a higher prevalence of brainstem lesions (24). These findings highlight the significance of C4 in the pathological mechanisms of NMO and its potential as a therapeutic target.

### 3.4 C6 activation in ON

C6 plays a pivotal role in the terminal pathway of the complement system, primarily through its contribution to MAC formation. The activation of C6 drives neuroinflammation and nerve damage by facilitating the assembly of MAC, which lyses cell membranes and exacerbates inflammatory responses (25). Research indicates that the absence or inhibition of C6 prevents MAC formation, thereby mitigating nerve damage and reducing inflammation (26). This underscores the therapeutic potential of targeting C6 to alleviate the pathological effects of ON and related neuroinflammatory conditions.

### 3.5 C7 activation in ON

C7 plays a crucial role in the pathological progression of ON by contributing to complement deposition in the optic nerve, which triggers demyelination—a key step in the disease process. Activation of C7 also leads to the upregulation of genes such as IRF7 and CXCL10, suggesting its involvement in regulating inflammatory responses (27). Furthermore, C7’s interaction with AQP4 antibodies exacerbates astrocyte injury, complement deposition, and demyelination while altering inflammation-related gene expression. These mechanisms collectively drive the inflammatory and degenerative pathology observed in ON, highlighting the significance of C7 as a therapeutic target.

### 3.6 C9 activation in ON

C9, the final component in forming of the membrane attack complex (MAC), is pivotal in cell lysis and tissue damage associated with optic neuritis (ON). During activation, C9 integrates into the C5b-8 complex, culminating in the insertion of the MAC into cell membranes. This process leads to cell destruction and exacerbates inflammation. Research indicates that limiting the incorporation of C9 into the MAC can effectively inhibit cell lysis (28). This finding highlights a potential therapeutic strategy to mitigate complement-mediated cytotoxicity and reduce neuronal and glial damage in ON.

## 4 Complement therapeutics in ON

Therapies targeting complement pathways aim to reduce inflammation, prevent tissue damage, and improve clinical outcomes in ON. Various complement inhibitors, either in development or clinical use, target different complement cascade components, offering the potential for effective intervention in ON and related conditions.

### 4.1 Inhibitors of complement activation

#### 4.1.1 C1q inhibitors

C1q inhibitors block the initiation of the classical complement cascade by preventing C1q from binding to its substrates, thereby reducing complement-mediated tissue damage, including CDC (29). ANX007, an anti-C1q antibody fragment, inhibits downstream C1q activity and has shown potential in minimizing retinal damage caused by classical complement activation. Preclinical studies suggest that ANX007 can significantly reduce tissue injury in complement-mediated retinal disorders (30). Beyond its role in CDC, C1q also contributes to neuroprotection by promoting neuronal survival and enhancing the expression of neurotrophic factors such as nerve growth factor (NGF) and neurotrophin-3 (NT-3), which support neuronal growth and maintenance (31). In NMOSD models, C1q-targeted monoclonal antibodies have been shown to reduce inflammatory demyelinating lesions caused by anti-AQP4 antibodies, highlighting their therapeutic potential in complement-mediated diseases (29). Early-phase studies of C1q inhibitors like ANX009 in healthy volunteers have demonstrated good safety and tolerability, supporting further exploration of C1q inhibitors in autoimmune diseases driven by complement activation (32). Advances such as C1qNb75 (33), a single-domain antibody targeting C1q, have also shown potential in inhibiting this pathway, offering promising therapeutic avenues.

#### 4.1.2 C3 inhibitors

C3 inhibitors represent a promising therapeutic approach for ON by targeting the central amplification point of the complement cascade, thereby preventing downstream activation across all pathways and reducing inflammation, tissue damage, and immune-mediated cytotoxicity. These inhibitors also address the inhibitory environment of the central nervous system (CNS) by inactivating the Rho signaling pathway, which impedes axonal regeneration. By inactivating Rho GTPase, C3 inhibitors facilitate neurite growth on inhibitory substrates like myelin-associated glycoprotein and myelin, enabling axonal repair (34). Pegcetacoplan, the first FDA-approved C3 inhibitor, blocks C3 cleavage, preventing the generation of active fragments C3a and C3b, thereby mitigating inflammation and reducing opsonization (35). This dual capacity to reduce complement-mediated damage and promote CNS repair makes C3 inhibitors a key area of interest in advancing ON treatment.

### 4.2 Inhibitors of terminal pathway activation

#### 4.2.1 C5 inhibitors

C5 inhibitors are a pivotal therapeutic class targeting the terminal pathway of the complement system by blocking C5 cleavage into C5a and C5b, thereby preventing the formation of the MAC responsible for cell lysis and inflammation. By halting the complement cascade at this critical juncture, C5 inhibitors effectively reduce inflammation, cell lysis, and complement-mediated cytotoxicity, making them particularly effective for complement-driven diseases like NMOSD. Eculizumab, the first C5 inhibitor approved for treating NMOSD, significantly reduces relapse rates but requires biweekly dosing, which can be burdensome for patients (36). Ravulizumab, a long-acting C5 inhibitor, addresses this limitation with dosing intervals extended to every 8 weeks (37). The CHAMPION-NMOSD trial[38] demonstrated that ravulizumab offers comparable efficacy to eculizumab while significantly improving patient convenience (38). However, challenges remain, including residual hemolytic activity and an increased risk of infections, particularly with encapsulated organisms like *Neisseria meningitidis*. To mitigate these risks, patients must be vaccinated against meningococci before starting treatment, and antibiotic prophylaxis may be recommended in high-risk scenarios. Despite these concerns, the sustained activity and improved convenience of long-acting C5 inhibitors underscore their transformative potential in managing complement-mediated diseases (39).

#### 4.2.2 Anaphylatoxin receptor blockers

Anaphylatoxin receptor blockers target the receptors for complement-derived inflammatory mediators C3a and C5a (C3aR and C5aR1), offering a therapeutic strategy to reduce inflammation and tissue damage while preserving the protective immune functions of the complement system. C3aR blockers primarily regulate immune cell recruitment and inflammatory amplification, with evidence suggesting a protective role for C3aR in conditions such as experimental meningococcal sepsis (40). Additionally, C5aR1 blockers are particularly effective in mitigating the pro-inflammatory and pathogenic effects of C5a. In NMOSD, modulation of C5aR1 activity can prevent astrocyte injury and demyelination caused by excessive complement activation. Age-related macular degeneration (AMD) studies have demonstrated that reducing C3a- and C5a-mediated inflammation preserves retinal integrity and slows degenerative processes (41). Avacopan, a C5aR antagonist approved for complement-mediated vasculitis, exemplifies this approach by selectively inhibiting C5a-driven inflammation while maintaining overall complement activity (42). Its success in vasculitis underscores the potential applicability of this strategy to optic neuritis and other complement-mediated disorders.

### 4.3 Complement regulators

#### 4.3.1 Recombinant regulators

Complement Factor H (CFH) is essential for regulating the alternative complement pathway, primarily by binding to C3b and acting as a cofactor for complement factor I (CFI) to inactivate C3b (43). Additionally, CFH accelerates the decay of the alternative C3 convertase, thereby controlling the complement system’s amplification loop caused by spontaneous C3b deposition. In NMOSD, approximately 9% of patients have autoantibodies against CFH, disrupting the interaction between CFH and C3b and leading to excessive complement activation and tissue damage (44). Similarly, CFH dysregulation in ON contributes to uncontrolled complement activity, resulting in inflammation, demyelination, and retinal RGC loss. Therapeutically, recombinant CFH or CFH-based treatments could restore proper complement regulation in patients with CFH deficiencies or autoantibodies. This targeted approach may mitigate tissue damage and inflammation, offering a promising neuroprotective strategy for complement-mediated diseases like ON and NMOSD. Further research into CFH-targeted therapies holds potential for advancing treatments in these conditions.

#### 4.3.2 Membrane-targeted regulators

Membrane-targeted regulators CD55 and CD59 are critical for protecting cells from complement-mediated damage. CD55, also known as the Decay-Accelerating Factor, inhibits C3 convertase formation, preventing the amplification of the complement cascade, while CD59, or Protectin, blocks the assembly of the MAC, protecting cells from lysis. These regulators are abundantly expressed in peripheral tissues, offering robust protection against complement-mediated cytotoxicity. However, their absence in the CNS renders CNS tissues particularly vulnerable to complement activation and AQP4-IgG-mediated damage, as observed in NMO (45). Therapeutically, enhancing CD59 expression in astrocytes has demonstrated neuroprotective effects, reducing demyelination and astrocyte loss in experimental models. Developing interventions that upregulate these regulators or mimic their protective functions could represent a novel strategy to mitigate complement-mediated damage in ON and other CNS disorders, offering new avenues for treatment and neuroprotection.

### 4.4 Plasma exchange

Plasma exchange (PLEX) involves physically removing circulating complement proteins, immune complexes, and autoantibodies. It has shown promise as a second-line treatment for severe ON, particularly when initial steroid therapies are ineffective. Studies demonstrate that PLEX can result in significant visual recovery for many patients. For instance, in a study involving 34 patients, over half experienced clinically meaningful improvements in visual function following PLEX treatment (46). Similarly, another study noted enhancements in visual acuity among patients with NMOSD who underwent PLEX after inadequate response to intravenous methylprednisolone (IVMP) (47).

### 4.5 Factor B and D inhibitors

Factor B is a serine protease that drives the proteolytic activity of the C3 and C5 convertases, forming the alternative pathway’s core amplification loop (48). Research has demonstrated that inhibiting Factor B effectively blocks the alternative pathway both *in vivo* and *in vitro*, preventing complement activation in serum. This highly selective and potent compound highlights its potential as a therapeutic agent for systemic treatment of complement-mediated diseases and supports its clinical development. Similarly, Complement Factor D (CFD) plays a critical role in the activation and amplification of the AP. CFD is a serine protease that serves as the rate-limiting enzyme, specifically cleaving Complement Factor B (CFB) to regulate complement activity (49).

### 4.6 MAC blockers

The activation of the terminal pathway of the complement system culminates in the formation of the MAC. Recent studies have identified CP010 as a novel therapeutic monoclonal antibody that specifically targets C6, effectively inhibiting MAC formation (26). CP010 achieves this by blocking the interaction between C6 and C5/C5b, thereby preventing MAC assembly. This mechanism suggests its potential therapeutic role in neurological conditions such as ON, highlighting the broader promise of MAC inhibitors in treating complement-mediated neurological diseases.

## 5 Challenges and future directions

The pathogenesis of optic neuritis (ON) is multifaceted, involving complement-dependent and complement-independent mechanisms, complicating therapeutic approaches (50). While complement inhibitors such as eculizumab have shown efficacy in reducing disease activity in NMOSD-associated ON, concerns persist regarding their long-term safety and effectiveness. Moreover, the precise role of complement inhibition within the broader therapeutic landscape remains uncertain, particularly given the availability of diverse treatments with distinct mechanisms of action. Additionally, the heterogeneity in disease presentation and progression among patients poses significant challenges to developing universally effective therapies (51).

Future directions in addressing ON focus on innovative, biomarker-driven, and tailored approaches to enhance treatment precision and efficacy. Combination therapies targeting both complement-dependent and complement-independent mechanisms may offer comprehensive disease management by improving therapeutic outcomes and minimizing adverse effects. Targeted modulation of specific complement proteins, such as C3 and C5, holds promise for controlling disease progression while maintaining essential immune functions. Moreover, advances in understanding the genetic and molecular underpinnings of ON facilitate personalized medicine, enabling customized interventions that align with individual patient profiles. These strategies aim to address the complex pathogenesis and heterogeneity of ON, optimizing treatment precision and efficacy (52).

## 6 Conclusion

The complement system is integral to the pathophysiology of ON, especially in AQP4-IgG and MOG-IgG-associated subtypes. Complement-targeted therapeutics present promising opportunities to reduce neuroinflammation and prevent tissue damage. As research and clinical trials continue to advance, they will provide deeper insights into the efficacy and safety of these treatments, ultimately refining therapeutic strategies and improving outcomes for patients with ON.
